# Effects of Cu, Ag, Zn, and Au Doping on the Electronic Structure and Optical Properties of *α*-Ga_2_O_3_: A Density Functional Theory Study

**DOI:** 10.3390/molecules31203763

**Published:** 2026-10-20

**Authors:** Wenyong Feng, Daojun Feng, Lele Jin, Qiangzhi Zhang, Dayu Xiao, Danfeng Zhu, Junbo Chen, Houjian Lin

**Affiliations:** 1 School of Physics and Electrical Engineering, Jiaying University, Meizhou 514015, China; fengwy26@mail2.sysu.edu.cn (W.F.); jinlele1804@163.com (L.J.); zhudanfeng@jyu.edu.cn (D.Z.); junbochen1991@163.com (J.C.); 2 Guangdong Hilly Agriculture Intelligent Equipment Engineering Technology Research Center, Jiaying University, Meizhou 514015, China; 3 School of Education, South China Normal University, Shanwei 516600, China; fengdaojun514@163.com

**Keywords:** *α*-Ga_2_O_3_, density functional theory calculations, metal doping, electronic structure, optical properties, p-type conductivity

## Abstract

*α*-Ga_2_O_3_ has attracted significant attention for high-power electronics and deep ultraviolet optoelectronics. Herein, density functional theory calculations were employed to systematically investigate the effects of metal doping, including Cu, Ag, Zn, and Au, on the structural stability, electronic structure, and optical properties of *α*-Ga_2_O_3_. The formation energy analysis reveals that all metal dopants preferentially substitute at the octahedral Ga sites, with lower formation energies under O-rich conditions compared to Ga-rich conditions, among which Zn doping exhibits the lowest formation energy under O-rich conditions, followed closely by Cu, indicating that both Zn and Cu can be readily incorporated into the *α*-Ga_2_O_3_ lattice. Metal doping introduces impurity levels within the band gap through the strong hybridization between dopant d orbitals and O 2p orbitals near the Fermi level, significantly narrowing the effective band gap and transforming the material from a pristine n-type to a p-type semiconductor. The charge density difference analysis further confirms the electron transfer from dopant metal atoms to neighboring O atoms, generating holes as the dominant charge carriers. Among the four dopants, Zn introduces the shallowest acceptor level near the valence band maximum, making it the most promising candidate for achieving effective p-type conductivity, while Cu, Ag, and Au create relatively deeper acceptor states. Furthermore, the optical properties demonstrate that metal doping causes a pronounced red shift in the absorption edge from the ultraviolet to the visible light region, as the impurity levels provide additional electronic transition pathways at lower photon energies. The enhanced visible light absorption, combined with the increased static dielectric constant and modified reflectivity spectra, indicates improved light-harvesting capability of the doped systems. These findings provide important theoretical guidance for the design of *α*-Ga_2_O_3_-based optoelectronic devices.

## 1. Introduction

Ga_2_O_3_ has garnered extensive research interest due to its outstanding physical and chemical properties, including a high breakdown electric field exceeding 8 MV/cm, excellent thermal stability, and high transparency in the deep ultraviolet region [1,2]. The exceptional Baliga’s figure of merit, which exceeds 3000 for *β*-Ga_2_O_3_, further underscores its superiority over traditional wide-band-gap semiconductors such as SiC and GaN for high-power applications [3,4]. To date, five different crystalline phases of Ga_2_O_3_ have been identified, namely *α*, *β*, *γ*, *ε*, and *δ* phases, among which the monoclinic *β*-Ga_2_O_3_ is the most thermodynamically stable phase and has been most extensively studied both experimentally and theoretically [5,6,7]. However, the metastable *α*-Ga_2_O_3_ phase with a corundum structure has recently attracted considerable attention due to its unique advantages over *β*-Ga_2_O_3_ [8]. Compared with *β*-Ga_2_O_3_, *α*-Ga_2_O_3_ possesses a wider direct band gap of approximately 5.3 eV, a larger Baliga’s figure of merit of about 3844, and a higher breakdown electric field ranging from 8 to 10 MV/cm [9,10]. Furthermore, *α*-Ga_2_O_3_ thin films and various heterojunctions have been successfully synthesized via different experimental techniques, including laser molecular beam epitaxy, mist chemical vapor deposition, halide vapor phase epitaxy, and metalorganic chemical vapor deposition [11,12,13,14]. The successful epitaxial growth of high-quality *α*-Ga_2_O_3_ films on commercially available sapphire substrates also highlights its great potential for practical device applications, particularly given the isostructural relationship between *α*-Ga_2_O_3_ and *α*-Al_2_O_3_, which facilitates lattice-matched epitaxy.

Despite these excellent properties, the development of *α*-Ga_2_O_3_-based devices faces a critical challenge: similar to *β*-Ga_2_O_3_, pristine *α*-Ga_2_O_3_ typically exhibits unintentional n-type conductivity due to the inevitable introduction of native point defects such as oxygen vacancies (V_O_), gallium interstitials (Ga_i_), and gallium vacancies (V_Ga_) during crystal growth [15]. Among these native defects, oxygen vacancies are widely recognized as the dominant source of n-type conductivity because they act as shallow donors, releasing free electrons into the conduction band [16]. This pristine n-type conduction seriously hinders the fabrication of p-n junctions, which are fundamental building blocks for most semiconductor devices, including light-emitting diodes, laser diodes, and solar cells. Therefore, achieving reliable p-type conductivity in *α*-Ga_2_O_3_ has become a longstanding research goal and is of paramount importance for advancing its practical applications in optoelectronics and power electronics. The fabrication of high-quality p-type Ga_2_O_3_ remains one of the most significant challenges in the field of ultra-wide-band-gap semiconductors.

Doping technology has been widely recognized as an effective approach to modulate the electronic and optical properties of wide-band-gap semiconductors [17,18]. Various dopant candidates have been explored for achieving p-type conductivity in Ga_2_O_3_, including group IA elements (Li, Na, K), group IIA elements (Mg, Ca), group IIB elements (Zn, Cd), and metals (Cu, Ag, Au, Ni) [19,20,21,22]. However, a comprehensive and systematic theoretical investigation comparing the effects of Cu, Ag, Zn, and Au doping on the structural, electronic, and optical properties of *α*-Ga_2_O_3_ within a single computational framework remains scarce. Several related studies have been reported, including the work by Pan on Cu, Ag, and Au doping in *α*-Ga_2_O_3_ [8], Wang et al. on Cu, Ag, and Au doping in *β*-Ga_2_O_3_ [22], and Zeng et al. on Cu and Zn doping in *α*-Ga_2_O_3_ [9]. The present work is the first to systematically compare all four dopants (Cu, Ag, Zn, Au) in *α*-Ga_2_O_3_ within a single computational framework, enabling direct comparison of acceptor-level depths and p-type doping tendencies.

The distinct crystal structure of *α*-Ga_2_O_3_ compared to *β*-Ga_2_O_3_ (monoclinic) may lead to different doping behaviors, defect formation energies, and electronic structures due to the different local coordination environments of Ga atoms. In the corundum structure, all Ga atoms are octahedrally coordinated by six O atoms, whereas *β*-Ga_2_O_3_ contains both octahedral and tetrahedral Ga sites. This structural difference is expected to influence the site preference, formation energy, and electronic behavior of metal dopants. Therefore, a comprehensive first-principles study is highly desirable to elucidate the doping mechanisms and their impacts on the material properties of *α*-Ga_2_O_3_. In this work, we employed first-principles calculations to systematically investigate the effects of Cu, Ag, Zn, and Au doping on the structural stability, electronic structure, and optical properties of *α*-Ga_2_O_3_. The formation energies, transition energy levels, band structures, density of states, charge density distributions, and optical absorption spectra were calculated and analyzed in detail. Our results provide valuable theoretical insights for designing p-type *α*-Ga_2_O_3_ materials and developing novel *α*-Ga_2_O_3_-based optoelectronic devices. We focus specifically on the corundum-structured *α*-Ga2O3 phase, where all Ga sites are crystallographically equivalent with octahedral coordination, providing a well-defined structural environment for studying doping mechanisms.

## 2. Results and Discussion

**Figure 1:**
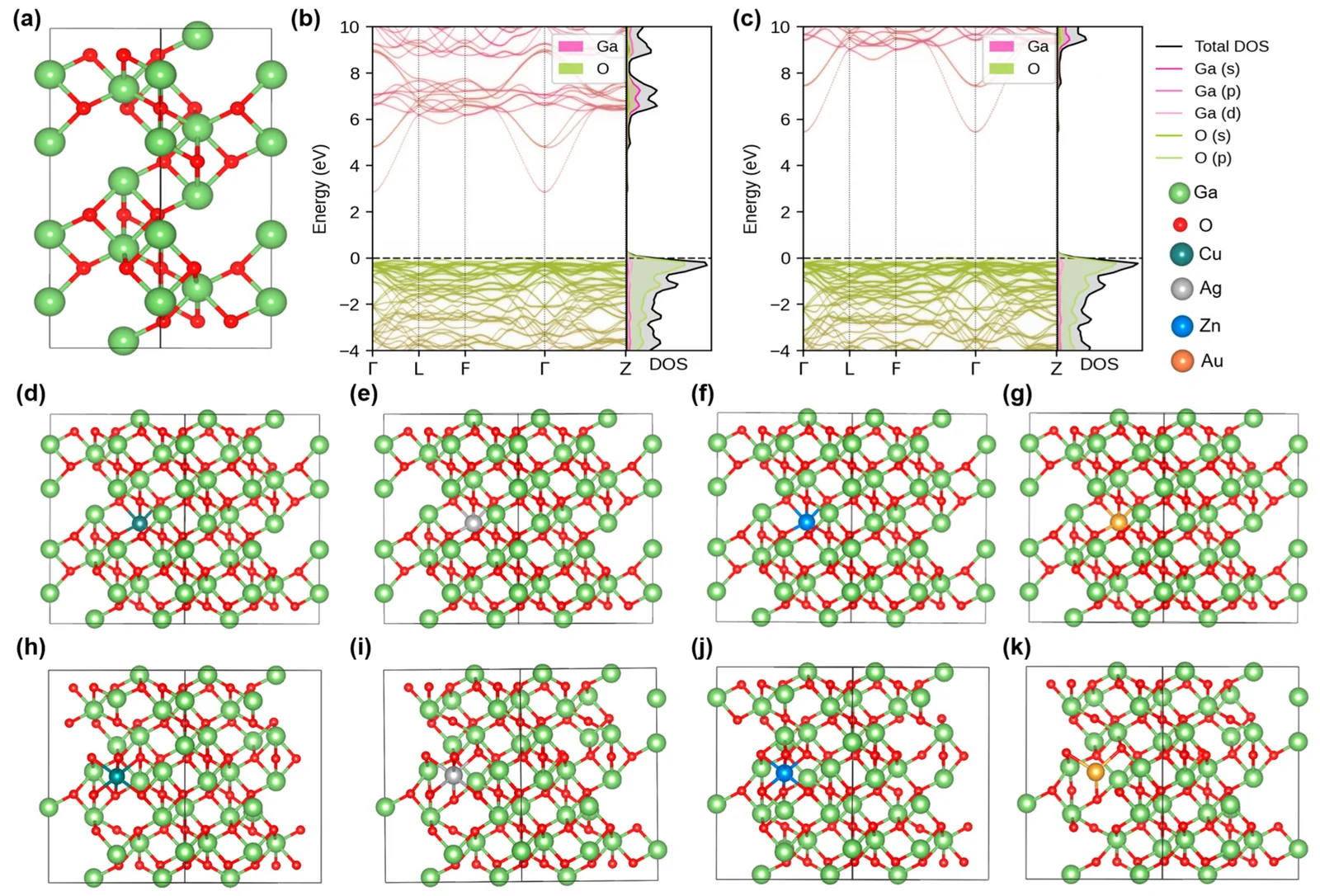
(**a**) The conventional structure and (**b**) electronic band structure of *α*-Ga_2_O_3_ based on the PBE functional. (**c**) Electronic band structure of *α*-Ga_2_O_3_ based on the HSE06 functional. The doped 2 × 2 × 1 supercell of (**d**) Cu_Ga_, (**e**) Ag_Ga_, (**f**) Zn_Ga_, (**g**) Au_Ga_, (**h**) Cu*_i_*, (**i**) Ag*_i_*, (**j**) Zn*_i_*, and (**k**) Au*_i_*.

The structural properties of *α*-Ga_2_O_3_ are explored, and its fully optimized conventional unit cell is illustrated in Figure 1. As a corundum-type phase, *α*-Ga_2_O_3_ crystallizes in the $R\bar{3}C$ symmetry, and its conventional cell contains six formula units of Ga_2_O_3_. For this hexagonal framework, only two independent lattice parameters, namely a = b and c, are required. Oxygen atoms adopt a hexagonal close-packed arrangement, while Ga atoms occupy two-thirds of the available octahedral interstitial sites. Each Ga-centered octahedron shares one face and three edges with three neighboring octahedra. Moderate distortion of these Ga-centered octahedra can be observed within the crystal lattice. Furthermore, electronic band structure calculations were carried out using both the PBE and HSE06 levels of theory, as illustrated in Figure 1b and Figure 1c, respectively. The calculated direct band gap is 2.55 eV at the Γ point, which is consistent with previous PBE calculations reported in the literature (typically 2.4–2.6 eV) but significantly underestimated compared to the HSE-calculated band gap(5.45) [23]. This well-known band gap underestimation is a common characteristic of the PBE functional arising from the incomplete cancellation of self-interaction and does not affect the qualitative conclusions of our comparative study. The VBM is predominantly composed of O 2p orbitals with minor contributions from Ga 3d and 4p orbitals, while the CBM consists mainly of Ga 4s orbitals with some Ga 4p admixture. The relatively flat valence band near the VBM indicates a large hole effective mass and consequently low hole mobility, which is consistent with experimental observations and explains the difficulty of achieving p-type conductivity in pristine Ga_2_O_3_. The conduction band is more dispersive, suggesting smaller electron effective masses and higher electron mobility.

When metal atoms are introduced into the *α*-Ga_2_O_3_ lattice, they preferentially substitute at the Ga sites rather than occupying interstitial positions, which is consistent with the lower formation energies for substitutional doping. The calculated models are displayed in Figure 1d–g. In the corundum structure of *α*-Ga_2_O_3_, all Ga sites are crystallographically equivalent and exhibit octahedral coordination. The optimized lattice parameters for the metal-doped systems show relatively small but systematic changes compared to the pristine material, as summarized in Table 1. For Cu-doped *α*-Ga_2_O_3_, the lattice constants remain nearly unchanged, with a negligible volume contraction of 0.05%. This can be attributed to the comparable ionic radii of Cu^+^ (0.60 Å) and Ga^3+^ (0.62 Å) in octahedral coordination. The Cu-O bond lengths in the doped system range from 1.94 Å to 1.99 Å, which are slightly shorter than the corresponding Ga-O bonds in the pristine material. Similarly, Zn-doped *α*-Ga_2_O_3_ exhibits minor lattice expansion, due to the slightly larger ionic radius of Zn^2+^ (0.74 Å) compared to Ga^3+^ (0.62 Å). The Zn-O bond lengths range from 1.98 Å to 2.03 Å, reflecting the expansion of the local coordination environment around the dopant. In contrast, Ag-doped and Au-doped systems show more pronounced lattice distortions because of the significantly larger atomic radii of Ag and Au atoms. For Ag-doped *α*-Ga_2_O_3_, the lattice parameters expand to a = 5.092 Å and c = 13.642 Å, corresponding to a volume expansion of 1.88%. The Ag-O bond lengths are elongated to 2.17 Å, indicating substantial local strain in the vicinity of the dopant. For Au-doped *α*-Ga_2_O_3_, the volume expansion reaches 2.48%, and the Au-O bond lengths extend to 2.21 Å. These structural modifications are closely related to the valence electron density and bonding characteristics of the dopant atoms, with larger and more electropositive dopants inducing greater lattice distortions.

Under O-rich growth conditions, the formation energies for Cu-doped and Zn-doped *α*-Ga_2_O_3_ are relatively low, indicating that Cu and Zn can be easily incorporated into the *α*-Ga_2_O_3_ lattice and that these doped systems possess high thermodynamic stability (Table 1). The relatively low formation energies suggest that the doping process is energetically favorable under O-rich conditions, and the formation of these defects is thermodynamically accessible. In contrast, Ag-doped and Au-doped systems exhibit high formation energies under O-rich conditions, suggesting that Ag and Au doping are energetically less favorable compared to Cu and Zn doping. Ag and Au dopants require non-equilibrium growth techniques or high-temperature annealing to achieve reasonable incorporation concentrations. The higher formation energies of Ag and Au can be attributed to the combined effects of ionic size mismatch (using ionic radii for the relevant oxidation states), oxidation state compatibility, and dopant–O bonding interactions. The interstitial formation energies under O-rich conditions are significantly higher, Cu*_i_* (3.85 eV), Ag*_i_* (5.12 eV), Zn*_i_* (2.95 eV), and Au*_i_* (6.45 eV), confirming the preferential substitution at Ga sites, and the interstitial structures are shown in Figure 1h–k. Our calculated lattice constants and formation energies agree well with the available theoretical values reported by Pan [8] (Cu, Ag, Au) and Zeng et al. [22] (Cu, Zn), confirming the reliability of the present computational framework.

**Figure 2:**
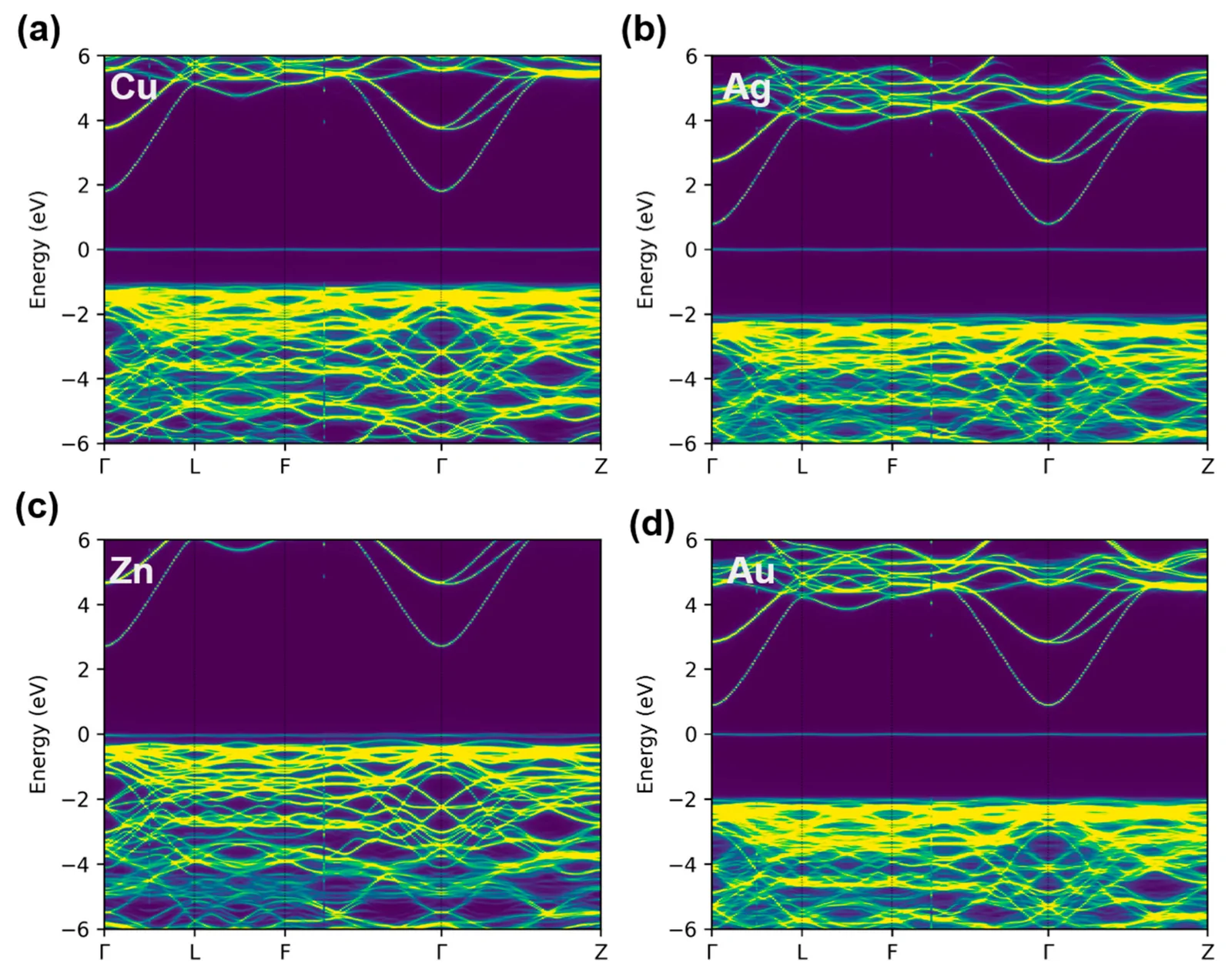
Electronic band structures of (**a**) Cu-doped, (**b**) Ag-doped, (**c**) Zn-doped, and (**d**) Au-doped *α*-Ga_2_O_3_. The band colors represent the orbital-projected character: cyan for the dopant d states (Cu 3d/Ag 4d/Zn 3d/Au 5d), yellow for O 2p states, and green for Ga s/p states. The Fermi level is set to 0 eV. The Fermi level is set to 0 eV.

Upon the introduction of metal dopants, significant modifications of the electronic band structure are observed, as shown in Figure 2a–d. All band structures and impurity level positions discussed below are from PBE calculations unless otherwise noted. For Cu-doped *α*-Ga_2_O_3_, the band gap is substantially reduced. An acceptor impurity level appears at approximately 0.85 eV above the VBM, primarily originating from the Cu 3d orbitals hybridized with neighboring O 2p states. This impurity level facilitates hole generation and p-type conductivity. Similarly, Ag doping introduces impurity levels near the VBM due to the strong interaction between Ag 4d and 5s electrons and O 2p electrons, reducing the band gap to 0.65 eV. The Ag-induced impurity levels are slightly deeper than those of Cu, as directly evidenced by the PDOS showing Ag 4d states appearing deeper in the valence band. This is connected to the longer Ag-O bonds and the different degrees of d–p hybridization. For Zn-doped *α*-Ga_2_O_3_, the band gap decreases to 2.43 eV, with a shallow acceptor level located very close to the VBM (approximately 0.02 eV above), suggesting that Zn is a promising candidate for achieving effective p-type doping with high hole concentration at room temperature. In the case of Au-doped *α*-Ga_2_O_3_, the band gap is reduced to 0.78 eV, with impurity levels introduced by Au 5d orbitals appearing both near the VBM and within the band gap, creating a more complex electronic structure.

The significant reduction in the band gap upon metal doping can be attributed to several interrelated mechanisms. First, the introduction of metal atoms creates additional electronic states within the band gap, which effectively narrows the energy separation between the valence and conduction bands by providing intermediate steps for electronic transitions. Second, the strong hybridization between metal d orbitals and O 2p orbitals leads to a redistribution of electron density near the Fermi level, promoting enhanced electron transitions between the VBM and CBM. According to band gap renormalization theory, the band gap narrowing in doped semiconductors is primarily governed by many-body effects arising from charge interactions and carrier-induced lattice relaxation, rather than the Burstein–Moss shift which typically widens the band gap in heavily doped n-type semiconductors. All metal-doped *α*-Ga_2_O_3_ systems exhibit narrower band gaps compared to the pristine material. The impurity bands induced by metal doping exhibit intermediate band (IB) characteristics, providing additional pathways for electron transitions and enabling the material to absorb lower-energy photons that would otherwise be transmitted through the pristine material. As mentioned above, PBE underestimates the band gap. The PBE band gap underestimation arises primarily from a downward shift in the conduction band minimum, whereas the valence band maximum and the dopant-derived acceptor levels are much less affected; consequently, the relative ordering of acceptor levels and the shallow/deep classification remain valid. The effective masses of electrons and holes at the band edges were also calculated from the curvature of the band structure near the Γ point. For pristine *α*-Ga_2_O_3_, the electron effective mass is calculated to be approximately 0.28 *m*_0_, while the hole effective mass is significantly larger at about 2.52 *m*_0_, confirming the asymmetric transport properties. Upon metal doping, the electron effective masses remain relatively unchanged, but the hole effective masses decrease substantially due to the increased dispersion of the valence band near the VBM induced by metal d and p orbital hybridization. This reduction in hole effective mass is beneficial for improving hole mobility and p-type conductivity. Among the doped systems, Zn-doped *α*-Ga_2_O_3_ exhibits the smallest hole effective mass (~0.82 *m*_0_), which correlates with its shallow acceptor level and makes it the most promising candidate for p-type applications.

**Figure 3:**
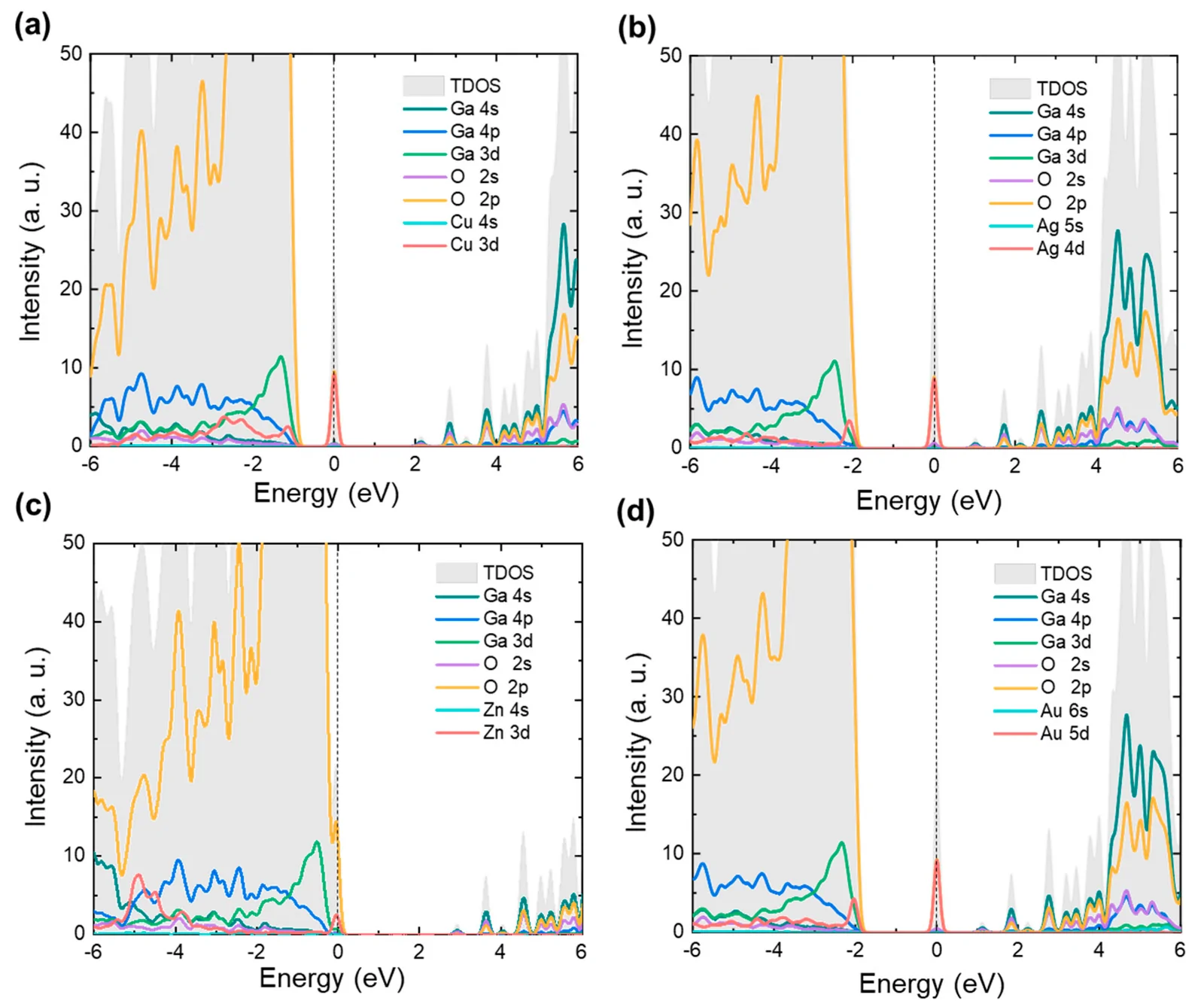
Total and partial density of states (TDOS and PDOS) for (**a**) Cu doped, (**b**) Ag doped, (**c**) Zn doped, and (**d**) Au doped *α*-Ga_2_O_3_.

To gain deeper insights into the electronic structure modifications induced by metal doping, the total density of states (TDOS) and partial density of states (PDOS) for the metal doped *α*-Ga_2_O_3_ systems were calculated and are shown in Figure 3. For Cu-doped *α*-Ga_2_O_3_, significant Cu 3d states appear near the Fermi level, indicating the formation of impurity levels within the band gap, as shown in Figure 3a. These Cu 3d states are strongly hybridized with the neighboring O 2p states, as evidenced by the overlapping peaks in the PDOS, forming covalent Cu-O bonds with partial ionic character. The partial occupation of the Cu 3d impurity levels (approximately one hole per Cu atom) suggests the presence of free holes, which is a characteristic feature of p-type doping. Similarly, Ag 4d and 5s states contribute to the impurity levels near the VBM in Ag-doped *α*-Ga_2_O_3_ [Figure 3b]. The Ag 4d states appear slightly deeper in the valence band compared to Cu 3d states, consistent with the deeper acceptor level of Ag doping. For Zn-doped *α*-Ga_2_O_3_, Zn 3d states are located slightly below the Fermi level at approximately −0.5 eV to 0 eV, forming a shallow acceptor level that is expected to significantly improve the carrier concentration [Figure 3c]. The Zn 3d-O 2p hybridization is weaker than that of Cu or Ag, which correlates with the less pronounced band gap reduction. In Au-doped *α*-Ga_2_O_3_, Au 5d states appear at Fermi level positions within the band gap above the VBM 2 eV, creating a deep impurity level.

**Figure 4:**
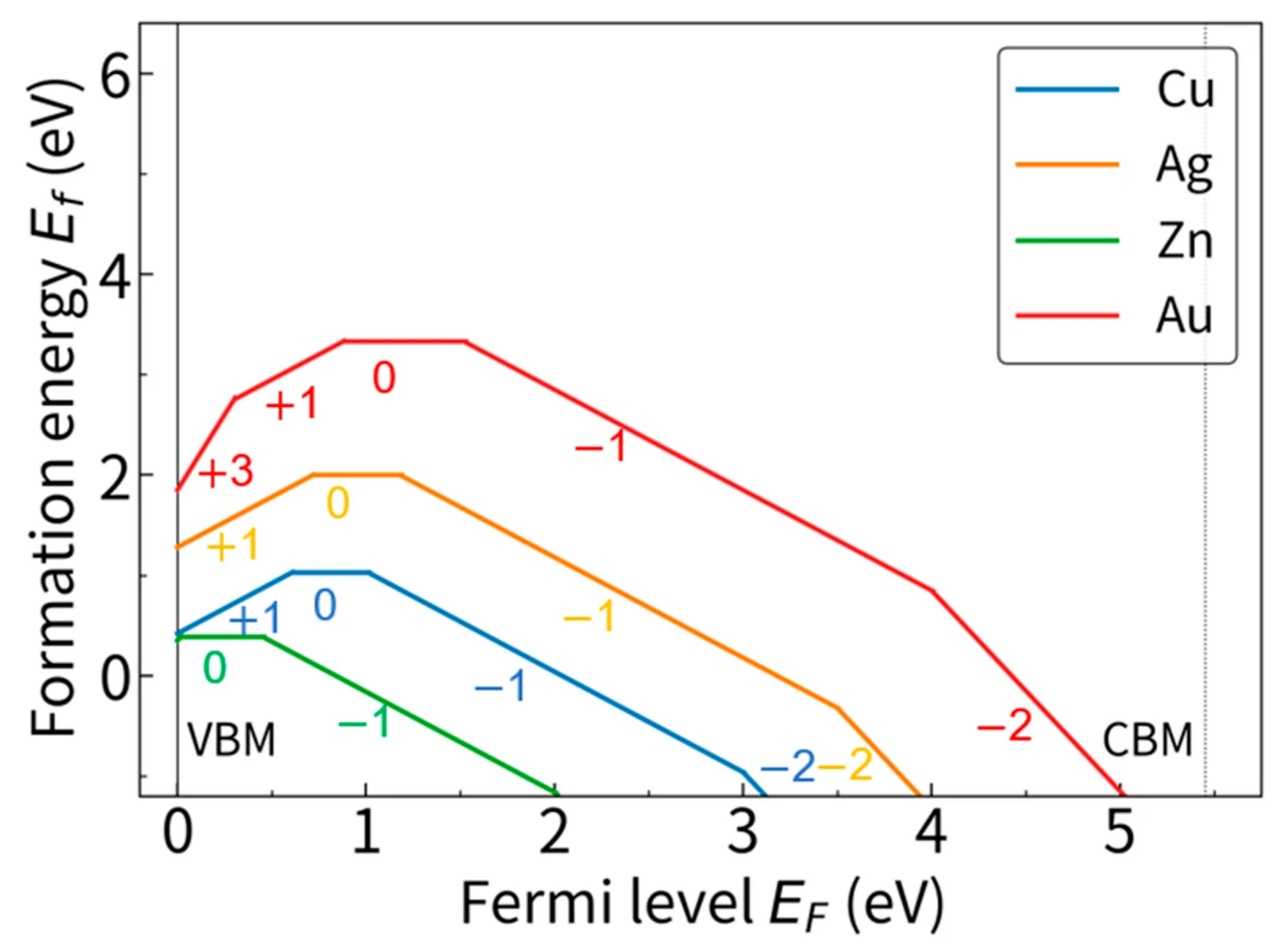
Formation energy as a function of Fermi level for Cu-, Ag-, Zn-, and Au-doped *α*-Ga_2_O_3_ under O-rich conditions. The solid black line on the left and the gray solid line on the right correspond to the VBM and the CBM, respectively.

The transition energy levels for the various charge states of the metal dopants were also calculated to determine their electrical activity, as shown in Figure 4. For Cu doping, the transition levels *ε*(+1/0) and *ε*(0/−1) are located at 0.61 eV and 1.01 eV above the VBM, respectively, indicating deep acceptor behavior. For Zn doping, the transition level *ε*(+2/0) occurs at only 0.02 eV above the VBM, indicating shallow acceptor characteristics that are highly desirable for p-type doping. These results strongly suggest that O-rich growth conditions are essential for achieving high-concentration metal doping in *α*-Ga_2_O_3_ and that Zn is the most promising candidate for shallow acceptor doping due to its extremely low transition levels near the VBM.

**Figure 5:**
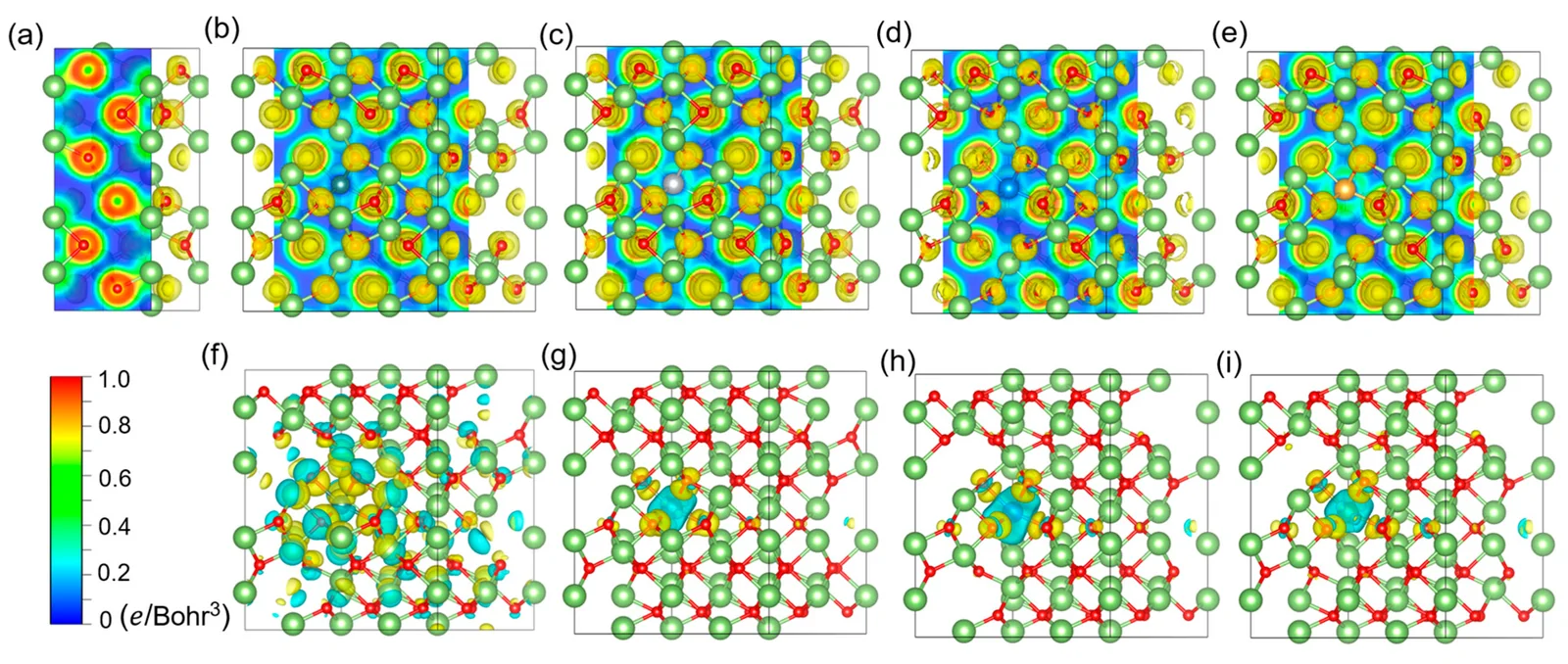
Electron localization functions (ELFs) of (**a**) *α*-Ga_2_O_3_ and *α*-Ga_2_O_3_ doped with (**b**) Cu, (**c**) Ag, (**d**) Zn and (**e**) Au. Charge density difference in *α*-Ga_2_O_3_ doped with (**f**) Cu, (**g**) Ag, (**h**) Zn and (**i**) Au.

The electron localization function (ELF), which quantifies the probability of finding an electron in the vicinity of a reference electron at a given point, provides direct insight into the bonding character and the degree of electron localization within the crystal. Figure 5a–e present the ELF distributions for pristine and metal-doped *α*-Ga_2_O_3_, where the color scale ranges from 0 (blue, completely delocalized) to 1.0 (red, perfectly localized), with ELF = 0.5 corresponding to the electron-gas-like reference. For pristine *α*-Ga_2_O_3_ [Figure 5a], high ELF values are predominantly localized around the O atoms, reflecting the localized nature of the O 2p lone pairs and core electrons. The Ga-O bonding regions exhibit relatively low ELF values, indicating a predominantly ionic bonding character with partial covalent contribution, which is consistent with the substantial electronegativity difference between Ga (1.81) and O (3.44) and with the flat valence band observed in the band structure [Figure 2a]. Upon metal doping [Figure 5b–e], the ELF distributions undergo pronounced modifications in the vicinity of the dopant sites. For Cu-doped *α*-Ga_2_O_3_ [Figure 5b], the ELF near the Cu site shows moderate localization, indicating that the Cu 3d electrons participate in bonding with neighboring O 2p states and form a hybridized bonding state with a partial covalent character. The relatively low electronegativity of Cu (1.90) compared to O (3.44) leads to a moderate degree of electron delocalization in the Cu-O bonding region. For Ag-doped *α*-Ga_2_O_3_ [Figure 5c], the ELF near the Ag site displays a more diffuse perturbation pattern compared to Cu, reflecting the larger atomic radius of Ag and the more spatially extended 4d orbitals. The Ag-O bonding region exhibits lower ELF values, suggesting weaker covalent bonding and a more ionic interaction, consistent with the larger lattice distortion (1.88% volume expansion) and higher formation energy (1.28 eV under O-rich conditions) of the Ag-doped system. For Zn-doped *α*-Ga_2_O_3_ [Figure 5d], strong electron localization is observed at the Zn site, corresponding to the fully filled 3d^10^ shell of Zn^2+^. The inert nature of the Zn 3d electrons results in minimal hybridization with the O 2p states, leading to the least perturbation of the electronic structure among all dopants. This is consistent with the shallowest acceptor level (0.02 eV above the VBM) and the weakest band gap reduction observed in the band structure analysis. For Au-doped *α*-Ga_2_O_3_ [Figure 5e], the strongest on-site electron localization is observed at the Au site, reflecting the relativistic contraction of the 6s orbital and the expansion of the 5d orbitals. The highly directional ELF distribution around Au indicates strong covalent character in the Au-O bonds, consistent with the high electronegativity of Au (2.54) and the complex impurity level architecture revealed by the PDOS analysis [Figure 3d].

The charge density difference (CDD), defined as Δ*ρ* = *ρ*(doped) − *ρ*(host) − *ρ*(metal _isolated_), provides an intuitive visualization of the electron redistribution upon metal doping. Figure 5f–i present the CDD isosurfaces for Cu-, Ag-, Zn-, and Au-doped *α*-Ga_2_O_3_, where yellow and cyan regions represent electron accumulation (Δ*ρ* > 0) and depletion (Δ*ρ* < 0), respectively. In all metal-doped systems, electron depletion is observed at the dopant sites, while electron accumulation occurs at the neighboring O atoms, indicating substantial charge transfer from the metal dopants to the surrounding oxygen atoms. This charge redistribution is consistent with the acceptor-type behavior of all four metal dopants, since Ga^3+^ donates three valence electrons to the lattice and the substitution by Cu^+^, Ag^+^, Zn^2+^, or Au^+^ generates holes as the dominant charge carriers, suggesting a tendency toward p-type behavior. The charge density difference analysis reveals electron depletion at the dopant sites and electron accumulation at the neighboring O atoms, indicating charge transfer from the dopant to the oxygen ligands. This is consistent with the acceptor-type behavior of the dopants. However, we note that charge redistribution around a dopant provides information about the local bonding character and charge transfer tendencies but does not by itself directly determine the sign or concentration of free carriers. The latter depends on the ionization of the acceptor levels and the compensation by other defects. Quantitative analysis using Mulliken population analysis reveals that the calculated positive charges for Cu, Ag, Zn, and Au dopants are +0.72, +0.79, +0.68, and +0.89, respectively, confirming that all metal dopants donate electrons to the surrounding O atoms and act as acceptors. The trend in charge transfer follows Au > Ag > Cu > Zn, which generally correlates with the electronegativity of the dopants (Au: 2.54, Ag: 1.93, Cu: 1.90, Zn: 1.65). The Bader charge analysis yields consistent trends, with charge transfer values of 1.26 *e* for Cu, 1.72 *e* for Ag, 1.66 *e* for Zn, and 2.02 *e* for Au, which are larger than the corresponding Mulliken values due to the different spatial partitioning scheme. The Bader charge transfer does not follow a simple monotonic trend with dopant electronegativity, as local coordination, bond lengths, and d–p hybridization strength also play important roles. Therefore, the correlation between the charge redistribution and the electronic structure is evident: dopants with more delocalized charge redistribution (Cu, Ag) introduce deeper impurity levels and larger band gap reductions, while Zn, with the most localized redistribution, introduces the shallowest acceptor level. This suggests that the degree of metal d orbital and O 2p hybridization, as directly visualized in both the ELF and CDD analyses, is the key factor governing the impurity level depth and the effectiveness of p-type doping in *α*-Ga_2_O_3_.

Understanding the optical response of semiconductor materials is indispensable for elucidating how they interact with electromagnetic radiation and for assessing their suitability for various optoelectronic applications. Within the framework of density functional theory, the frequency-dependent dielectric function can be computed from first principles, yielding both the real and imaginary components from which the absorption coefficient and other optical parameters are subsequently derived. The underlying formalism rests on the dipole approximation for direct interband transitions and the Kramers–Kronig dispersion relations, which link the dispersive and dissipative parts of the dielectric response. A comprehensive derivation of the relevant equations is available in standard solid-state physics references [24]. The frequency-dependent complex dielectric function is expressed as:

(1) $$\begin{array}{c} \varepsilon \left(\omega \right)=\varepsilon_{1}\left(\omega \right)+i\varepsilon_{2}\left(\omega \right) \end{array},$$

Here, *ε*_1_(*ω*) denotes the real part associated with material energy-storage characteristics, whereas *ε*_2_(*ω*) corresponds to optical energy dissipation and light absorption. The imaginary component *ε*_2_(*ω*) is governed by momentum-matrix elements connecting occupied and unoccupied electronic wavefunctions. The absorption coefficient *α*(*ω*) can be further evaluated from the real and imaginary dielectric components *ε*_1_(*ω*) and *ε*_2_(*ω*). The corresponding expressions are as follows [25]:

(2) $$\begin{array}{c} \varepsilon_{2}\left(\omega \right)=\frac{c}{\omega^{2}}\sum_{V,C}\int_{\mathrm{BZ}}\frac{2}{{\left(2\pi \right)}^{2}}{\left|M_{C,V}\left(\kappa \right)\right|}^{2}\times \delta \left(E_{C}^{\kappa }-E_{V}^{\kappa }-\hbar \omega \right)d^{3}\kappa \end{array},$$

In Equation (2), *c* denotes the speed of light; the subscripts *C* and *V* refer to conduction-band and valence-band electronic states; *BZ* stands for the first Brillouin zone; ${\left|M_{C,V}\left(\kappa \right)\right|}^{2}$ is the momentum-matrix element between electronic wavefunctions; *κ* represents the wave vector; *ω* is the angular frequency of incident light; $E_{C}^{\kappa }$ and $E_{V}^{\kappa }$ are the respective eigenvalues of conduction-band and valence-band states; and *ħ* is the reduced Planck constant.

(3) $$\begin{array}{c} \varepsilon_{1}\left(\omega \right)=1+\frac{2}{\pi }\rho_{0}\int_{0}^{\infty }\frac{\omega^{\prime }\varepsilon_{2}\left(\omega \right)}{\omega^{\prime 2}-\omega^{2}}d\omega^{\prime } \end{array},$$

(4) $$\begin{array}{c} \alpha \left(\omega \right)=\sqrt{2}{\left[\sqrt{\varepsilon_{1}^{2}\left(\omega \right)+\varepsilon_{2}^{2}\left(\omega \right)}-\varepsilon_{1}\left(\omega \right)\right]}^{1/2} \end{array}.$$

In the above equations, *ρ*_0_ denotes the Cauchy principal value for the integral. The static dielectric constant *ε*(0) refers to the real part of the electronic dielectric function *ε*_1_(*ω*) evaluated at zero photon energy, capturing the electronic contribution only; ionic contributions from lattice vibrations are not included.

**Figure 6:**
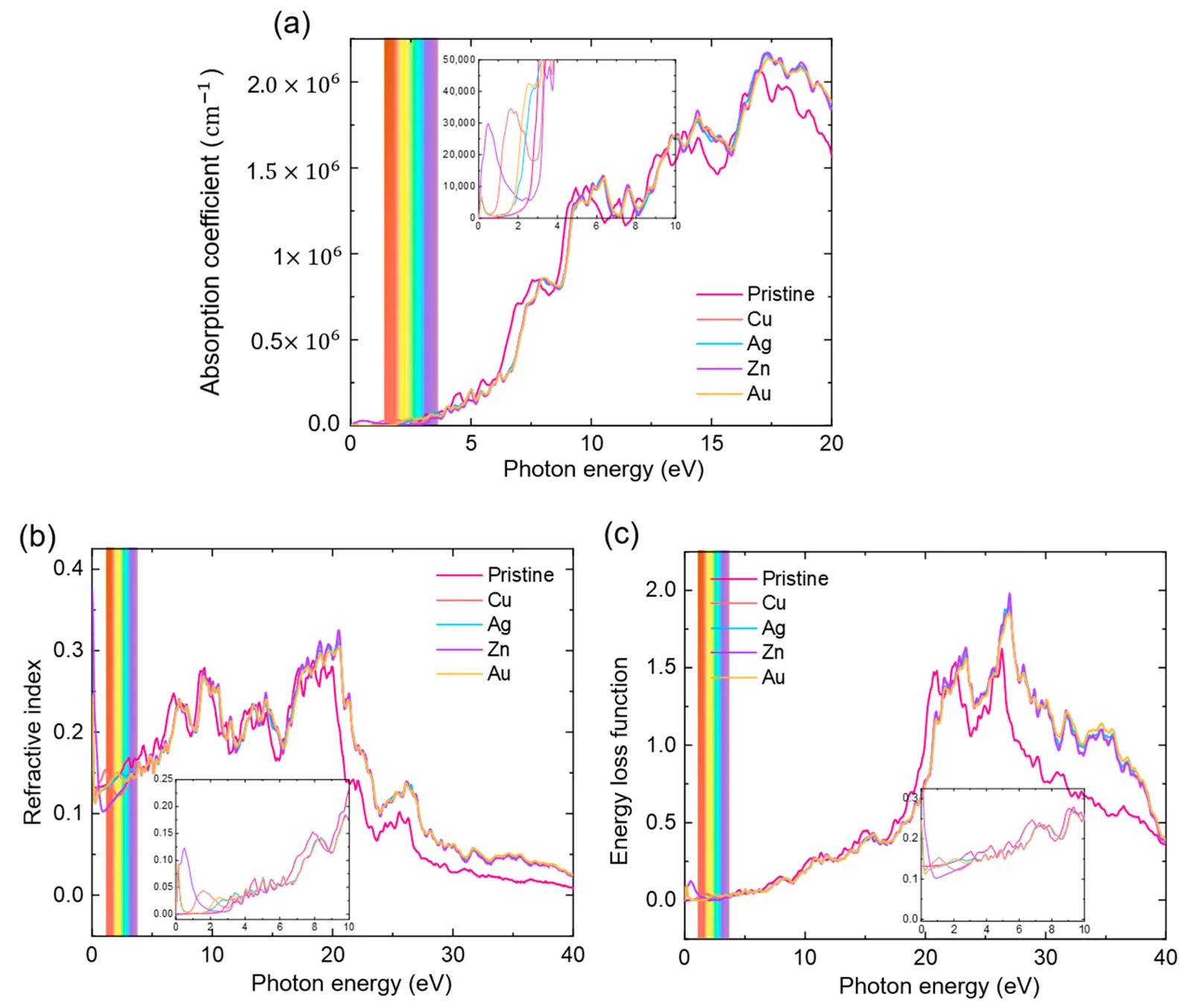
Intrinsic and metal-doped *α*-Ga_2_O_3_: (**a**) absorption spectra, (**b**) reflectivity spectra and (**c**) loss functions.

The calculated optical absorption coefficients of pristine and metal-doped *α*-Ga_2_O_3_ as a function of photon energy are displayed in Figure 6a. For pristine *α*-Ga_2_O_3_, the absorption spectrum exhibits strong absorption peaks at approximately 7.5 eV, 9.7 eV, 14.5 eV, and 17.0 eV in the ultraviolet region, which are associated with the interband electronic transitions from the O 2p valence states to the Ga 4s and 4p conduction states. This strong UV absorption characteristic is consistent with the ultra-wide-band-gap nature of *α*-Ga_2_O_3_ and confirms its potential for deep-UV photodetector applications. The absorption edge near 2.5 eV corresponds to the fundamental band gap, and the relatively weak absorption below this energy confirms the high transparency of pristine *α*-Ga_2_O_3_ in the visible and infrared regions. Upon metal doping, significant modifications of the optical absorption spectra are observed across the entire photon energy range studied. In the high-energy UV region above 6 eV, the absorption coefficients of all metal-doped systems show similar overall profiles to the pristine material, but within the 6–10 eV region, there are slightly reduced peak intensities, while above 10 eV, there are slightly enhanced intensities at the main absorption peaks. This indicates that the pristine UV absorption capability of *α*-Ga_2_O_3_ is largely preserved after metal doping, which is important for maintaining its deep-UV detection performance. The slight reduction in UV absorption intensity can be attributed to the dilution effect of the Ga-O bonds by the metal dopants and the modification of the conduction band structure. More importantly, in the low-energy region (visible and near-infrared), new absorption peaks emerge for all metal-doped systems, which are entirely absent in the pristine material. For Cu-doped *α*-Ga_2_O_3_, new absorption peaks appear at approximately 1.2 eV and 2.5 eV, corresponding to electronic transitions from the O 2p states to the Cu 3d impurity states and between the induced impurity levels (inter-hole transitions), respectively. Similarly, Ag-doped *α*-Ga_2_O_3_ exhibits new absorption features at approximately 3.0 eV, while Au-doped *α*-Ga_2_O_3_ shows absorption peaks at approximately 2.5 eV. For Zn-doped *α*-Ga_2_O_3_, a relatively weak but distinct absorption peak appears near 0.8 eV in the infrared region, corresponding to transitions from the O 2p valence band to the shallow Zn 3d acceptor level. The appearance of these new absorption features in the visible and infrared regions is a direct consequence of the metal-induced impurity levels within the band gap, which provide low-energy pathways for electronic transitions. The red shift in the absorption edge from the UV to the visible light region can be quantified by examining the photon energy at which the absorption coefficient reaches a threshold value of 10^4^ cm^−1^. For pristine *α*-Ga_2_O_3_, this threshold occurs at approximately 2.5 eV. For the metal-doped systems, the threshold shifts to lower energies. This enhancement in visible light absorption is particularly important for transparent conductive oxides applications, where efficient harvesting of the solar spectrum requires strong absorption across both the UV and visible regions.

Optical spectra were calculated using the PBE functional within the independent-particle approximation. The PBE band gap underestimation shifts all spectral features to lower energies. The pristine absorption edge at ~2.5 eV corresponds to the PBE band gap, not the experimental 5.3 eV. The reflectivity spectra and energy loss functions for pristine and metal-doped *α*-Ga_2_O_3_ are presented in Figure 6b and Figure 6c, respectively. The reflectivity *R*(*ω*) describes the fraction of incident light that is reflected from the material surface and is important for applications involving optical cavities and waveguides. In the deep UV region above 15 eV, all systems exhibit similar reflectivity characteristics with prominent peaks corresponding to plasmon resonances and interband transitions. In the near-infrared, visible, and ultraviolet regions (1.5–6 eV), metal-doped systems show enhanced reflectivity compared to the pristine material, which may be advantageous for back-reflector applications in photodetector configurations, though high reflectivity at an emitting surface can be detrimental to optical outcoupling in LED or laser devices. The enhanced visible reflectivity is also consistent with the metallic-like character introduced by the partially filled metal d states near the Fermi level. The energy loss function *L*(*ω*) = *Im*[−1/*ε*(*ω*)] quantifies the energy dissipation of fast electrons traversing the material and provides information about collective excitations such as plasmons. For pristine *α*-Ga_2_O_3_, the energy loss function displays prominent peaks at approximately 12.5 eV, 15.0 eV, and 21.5 eV in the extreme ultraviolet region, corresponding to bulk plasmon excitations and interband transitions. The introduction of metal dopants leads to the appearance of new loss features in the visible region (1–3 eV). Low-energy features in the loss function are associated with interband transitions involving the TM d-derived impurity states, which appear as modifications to the low-energy dielectric response. These low-energy loss features correlate with the impurity-related absorption peaks and further confirm the significant modification of the electronic structure by metal doping. The energy-loss functions are remarkably similar because *L*(*ω*) is primarily governed by the bulk plasmon of the host lattice at 12–22 eV, far above the dopant-induced transitions. The intensity of the plasmon peaks in the UV region is slightly reduced in the doped systems, consistent with the dilution of the Ga-O bonding network.

## 3. Computational Methods

All first-principles calculations in this study were performed using the QUANTUM ESPRESSO code based on density functional theory (DFT) [26,27]. The exchange-correlation interactions were treated within the generalized gradient approximation (GGA) parameterized by the Perdew–Burke–Ernzerhof (PBE) functional [28]. In addition, the Heyd–Scuseria–Ernzerhof (HSE06) hybrid functional with a Fock-exchange mixing parameter *α* = 0.35 and a screening parameter *μ* = 0.21 Å^−1^ was employed solely as a benchmark to verify that the PBE-calculated band structure is consistent with the more accurate hybrid-functional results; this parameter set has been reported to reproduce the experimental band gap of *α*-Ga_2_O_3_ (5.46 eV) [29]. The projector-augmented wave (PAW) pseudopotentials were employed to describe the ion–electron interactions, which provide accurate descriptions of the electronic structure while maintaining computational efficiency [30,31]. The PAW pseudopotentials employed in this study inherently include scalar relativistic effects at the pseudopotential generation stage, which is particularly important for the heavy dopants Ag and Au. A plane-wave basis set with a kinetic energy cutoff of 450 eV was adopted, which was verified to be sufficient for converging the total energy to within 1 meV/atom. The energy convergence criterion for the self-consistent field calculations was set to 1 × 10^−5^ eV/atom. All atomic positions and lattice parameters were fully relaxed until the residual forces on each atom were less than 0.01 eV/Å. For the structural modeling, a 2 × 2 × 1 supercell of *α*-Ga_2_O_3_ containing 48 Ga atoms and 72 O atoms was constructed based on the optimized primitive cell. One Ga atom was substituted by a metal dopant (Cu, Ag, Zn, or Au), corresponding to a doping concentration of approximately 2.08%. This supercell size provides a reasonable balance between computational accuracy and efficiency for studying isolated point defects. The Brillouin zone integration was performed using Monkhorst-Pack *k*-point grids of 4 × 4 × 2 for structural relaxation and self-consistent calculations, and a denser grid of 9 × 9 × 4 was employed for the calculations of density of states (DOS) and optical properties to ensure accurate electronic structure representations. Spin-polarized calculations were performed for all doped systems. All production calculations, including structural optimization, formation energies, electronic band structures, density of states, effective masses, and optical properties were performed using the PBE functional. The HSE06 functional was used only as a benchmark to confirm that the PBE band structure agrees with the hybrid-functional results. Spin-orbit coupling (SOC) effects were not included in the main calculations. Test calculations with SOC for the Au-doped system showed that SOC splits the degenerate d-orbital-derived impurity states but does not alter the qualitative picture of acceptor-type impurity levels. The band gap modification due to SOC is approximately 0.1–0.2 eV.

The defect formation energy *E_f_* for metal M doping in *α*-Ga_2_O_3_ in charge state q was calculated using the standard formalism established in the literature [32,33]. The formation energy is given by:

(5) $$E_{f}(\epsilon )=E_{\mathrm{def}}(\epsilon ,q)-E_{\mathrm{pristine}}(\epsilon ,q)\pm \sum_{i}n_{i}\mu_{i}(\epsilon )+q(E_{v}(\epsilon )+\mu_{e})+E_{\mathrm{corr}},$$

where *E_def_*(*ϵ*,*q*) and *E_pristine_*(*ϵ*,*q*) are the total energies of the doped and perfect supercells, respectively; *n_i_* is the number of atoms added to (*n_i_* > 0) or removed from (*n_i_* < 0) the perfect supercell; *μ_i_* is the chemical potential of the corresponding atom, representing the energy of the reservoir with which atoms are exchanged; *μ_e_* denotes the electronic chemical potential, and *E_v_*(*ϵ*) represents the strain-modulated energy level of the valence band maximum (VBM). The *E_corr_* term acts as a systematic correction, which is implemented to remove spurious Coulomb interactions between charged defects that arise from periodic boundary conditions within supercell calculations. The finite-size correction term *E_corr_* was implemented using the Freysoldt–Neugebauer–Vande Walle (FNV) charge correction scheme [29,34,35]. For charged defects, The FNV scheme employs the static dielectric constant (ε = 10.2) of *α*-Ga_2_O_3_ together with a potential alignment correction to account for both the monopole electrostatic energy and the band edge misalignment [36]. For the 2 × 2 × 1 supercell, the residual error after FNV correction is expected to be below 0.1 eV for charge states up to *q* = ±2, ensuring reliable, charged defect formation energies and transition levels. The chemical potentials satisfy the boundary conditions 2*μ_Ga_* + 3*μ_O_* = $\mu_{{\mathrm{Ga}}_{2}O_{3}}$, where $\mu_{{\mathrm{Ga}}_{2}O_{3}}$ is the chemical potential of bulk *α*-Ga_2_O_3_. Two extreme growth conditions were considered: Ga-rich and O-rich conditions. Under O-rich conditions, the chemical potential of oxygen is determined from the total energy of an O_2_ molecule, while under Ga-rich conditions, the chemical potential of Ga is obtained from the energy of bulk Ga metal. The chemical potentials of the dopant atoms were referenced to their metallic phases: *μ*_cu_ from fcc Cu metal, *μ*_Ag_ from fcc Ag metal, *μ*_Zn_ from hcp Zn metal, and *μ*_Au_ from fcc Au metal. Under O-rich conditions, the upper bound of each dopant chemical potential is constrained by the formation of the respective oxide phase (Cu_2_O, Ag_2_O, ZnO, Au_2_O_3_). The transition energy level between charge states was determined by the Fermi level position where the two charge states have equal formation energies, which corresponds to the thermal ionization energy of the defect.

Effective masses were calculated by fitting the band dispersion near the Γ point using a parabolic approximation along three principal crystallographic directions: Γ → M, Γ → K (in-plane), and Γ → A (along c). The fitting range was ±0.05 Å^−1^ around Γ. Mulliken population analysis was performed using the pseudoatomic orbital reconstruction method. We note that Mulliken charges are basis-set-dependent; Bader charge analysis, which is based on the topological analysis of the charge density and is independent of the basis set, is considered more reliable for plane-wave calculations and is presented as the primary quantitative charge analysis. Mulliken population analysis was performed using the pseudoatomic orbital reconstruction method with CP2K code [37]. We note that Mulliken charges are basis-set-dependent; Bader charge analysis, which is based on the topological analysis of the charge density and is independent of the basis set, is considered more reliable for plane-wave calculations and is presented as the primary quantitative charge analysis.

## 4. Conclusions

In summary, first-principles calculations demonstrate that Cu, Ag, Zn, and Au dopants preferentially substitute at octahedral Ga sites in *α*-Ga_2_O_3_, with Cu and Zn exhibiting the lowest formation energies under O-rich conditions. All four dopants introduce acceptor-type impurity levels, suggesting a tendency toward p-type behavior by introducing acceptor levels through metal d and O 2p hybridization. The key finding is that the acceptor level depth is governed by the degree of d and p hybridization. The dopant Zn, with its filled 3d shell and weakest hybridization, produces the shallowest acceptor (0.02 eV above VBM) and the smallest hole effective mass, making it the optimal p-type dopant; Cu, Ag, and Au introduce progressively deeper levels. Metal doping also red-shifts the absorption edge into the visible region while preserving the intrinsic deep-UV response, broadening the optoelectronic application window. We note that this study considers isolated substitutional dopants and does not account for compensation by native donor defects. However, these results establish d and p orbital hybridization strength as the key descriptor for rational p-type doping design in *α*-Ga_2_O_3_.

## Figures and Tables

**Table 1:** The optimized lattice parameters and formation energies (eV) for pristine and metal-doped *α*-Ga_2_O_3_.

System	a (Å)	c (Å)	V (Å^3^)	*E_f_* (O-Rich)	*E_f_* (Ga-Rich)	Ionic Radius (Å)
Pristine	5.055	13.586	1202.66	—	—	—
Cu-doped	5.056	13.574	1202.03	0.42	6.02	0.74
Ag-doped	5.092	13.642	1224.85	1.28	7.35	1.15
Zn-doped	5.059	13.583	1204.15	0.35	5.62	0.73
Au-doped	5.098	13.658	1231.42	1.85	7.92	1.37

## Data Availability

The original contributions presented in this study are included in the article. Further inquiries can be directed to the corresponding author.
